# Observing microscopic structures of a relativistic object using a time-stretch strategy

**DOI:** 10.1038/srep10330

**Published:** 2015-05-28

**Authors:** E. Roussel, C. Evain, M. Le Parquier, C. Szwaj, S. Bielawski, L. Manceron, J.-B. Brubach, M.-A. Tordeux, J.-P. Ricaud, L. Cassinari, M. Labat, M.-E Couprie, P. Roy

**Affiliations:** 1 Laboratoire PhLAM, UMR CNRS 8523, Université Lille 1, Sciences et Technologies, 59655 Villeneuve d’Ascq, France; 2 Centre d’Etudes et de Recherches Lasers et Applications (CERLA), 59655 Villeneuve d’Ascq, France; 3 Synchrotron SOLEIL, L’Orme des Merisiers, Saint-Aubin, BP 48, 91192 Gif-sur-Yvette Cedex, France

## Abstract

Emission of light by a single electron moving on a curved trajectory (synchrotron radiation) is one of the most well-known fundamental radiation phenomena. However experimental situations are more complex as they involve many electrons, each being exposed to the radiation of its neighbors. This interaction has dramatic consequences, one of the most spectacular being the spontaneous formation of spatial structures inside electrons bunches. This fundamental effect is actively studied as it represents one of the most fundamental limitations in electron accelerators, and at the same time a source of intense terahertz radiation (Coherent Synchrotron Radiation, or CSR). Here we demonstrate the possibility to directly observe the electron bunch microstructures with subpicosecond resolution, in a storage ring accelerator. The principle is to monitor the terahertz pulses emitted by the structures, using a strategy from photonics, time-stretch, consisting in slowing-down the phenomena before recording. This opens the way to unpreceeded possibilities for analyzing and mastering new generation high power coherent synchrotron sources.

Interaction of a relativistic electron bunch with its own created electromagnetic field can lead to the so-called *microbunching instability*. It is encountered in systems based on linear accelerators^1^, solar flares^2^,^3^,^4^, as well as in the widely-used storage rings facilities^5^,^6^,^7^,^8^,^9^,^10^,^11^,^12^,^13^,^14^ (synchrotron radiation facilities), where electron bunches are forced to circulate onto a closed loop trajectory. Above a threshold electron bunch density, a longitudinal modulation or *pattern* appears with a characteristic period at the millimeter or sub-millimeter scale^5^,^6^,^7^,^15^. This structure emits intense pulses of terahertz radiation (typically more than 10000 times normal synchrotron radiation), called Coherent Synchrotron Radiation (CSR). Each CSR pulse shape may be viewed as an “image” of the electron bunch microstructure.

As a consequence, a particularly efficient way to study this fundamental physical effect consists in using existing *user-oriented* storage rings (i.e., synchrotron radiation facilities). Indeed, recording CSR pulses emitted at each turn in such a storage ring would be theoretically sufficient to follow the electron microstructure evolution over a long time. This has been recently demonstrated in a special case where the microstructure wavelength is in the centimeter range and CSR emission occurs in the tens of GHz range^16^, thus being accessible to conventional electronics. However, in most storage rings such as ALS^6^, ANKA^10^, BESSY^7^, DIAMOND^11^,^12^, ELETTRA^11^, or SOLEIL^14^, etc., the CSR emission occurs at frequencies that are so high (above 300 GHz for the present case of SOLEIL) that no suitable recording electronics is available at the moment, nor expected in the near future.

Here, we propose a strategy that overcomes these limitations, and thus enables such fundamental studies in many storage ring facilities. It consists in “slowing down” the signals so that they can be recorded by conventional oscilloscopes (Fig. 1). This is a two-step process as shown in Fig. 2. First, the THz CSR pulse is encoded into a laser pulse, using the well established technique of THz electro-optic sampling^10^,^17^,^18^,^19^. Then the key point is to use a setup based on the so-called *photonic time-stretch* strategy^20^,^21^,^22^, which consists in dispersing the pulses in a long fiber. Under some condition on the fiber length *L*_1_, the output pulse is a replica of the original signal, except that is slowed down by a magnification factor (or stretch factor)^23^:


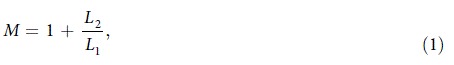


where *L*_1_ and *L*_2_ are the input and output fiber lengths (*L*_1_ = 10.7 m and *L*_2_ = 2 km for the results presented hereafter, leading to a stretch factor *M* = 190). Using such a strategy instead of the classical *spectral encoding*^17^,^18^,^19^ method presents two advantages: (i) a much higher acquisition rate (i.e., the number of recorded terahertz pulses per second), which is directly linked to the laser repetition rate, and (ii) the possibility of performing balanced detection^24^, a crucial point for reaching high sensitivity.

The time-stretch THz recording system (detailed in the Methods section) was able to acquire 88 × 10^6^ terahertz pulses per second, and its sensitivity was measured to be 37 V/cm inside the crystal (see Methods). This allowed us to record the terahertz CSR pulses (electric field, including envelope and carrier) emitted at each turn (i.e., every 1.2 *μ*s) at the AILES beamline of the SOLEIL storage ring.

First experiments with the time-stretch acquisition setup allowed us to record the THz CSR for each revolution in the ring, and to visualize the predicted microstructure in the electron bunch circulating at Synchrotron SOLEIL. The structure is clearly visible in Fig. 3, which represents a typical series of individual pulses, recorded at successive round-trips.

Furthermore, the new type data thus obtained contain extremely detailed information on the long-term evolution of the structures. In order to summarize the dynamical features, we displayed the pulse evolution of Fig. 3 as a colormap versus the longitudinal coordinate, and the number of turns in the storage ring (Fig. 4). It clearly appears that—though their evolutions are very complex—the structures are constituted of oscillations with a characteristic wavenumber in the millimeter range (more precisely 10 cm^−1^, or 0.3 THz as shown in the inset of Fig. 3). This is consistent with previous indirect observations, by spectrometric measurements, of a strong terahertz emission peak at 10 cm^−1^ (Ref. 14). Moreover, the recordings (as in Fig. 4) systematically revealed complex drifting evolution during the revolutions, that remind the ubiquitous irregular behaviors that occur in fluid dynamics^25^. We believe that this new detailed data will provide a real platform for testing and refining theoretical models of relativistic electron bunch dynamics.

Several important features of the experimental observations can be reproduced with already existing theoretical models^5^,^14^,^26^,^27^, where only longitudinal dynamics of the electrons is taken into account. Each electron *i* is characterized by its instantaneous position *z*_*i*_ and energy variable *δ*_*i*_. A map (at each turn *n*) can then be written for the evolutions of 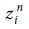 and 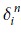. Taking the continuous limit for the number of round-trips *n*^28^,^29^:


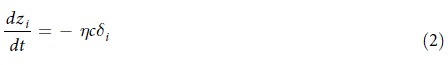



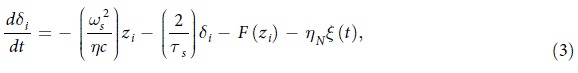


where *t* is a continuous time associated to the number of round-trips *n*. 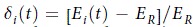, with *E*_*i*_(*t*) the electron energy, and *E*_*R*_ the reference energy corresponding to the synchronous electron (2.75  GeV here). *F*(*z*_*i*_) characterizes the electric field at position *z*_*i*_ created by the whole electron bunch, and is the main ingredient of the instability. We use here the same form as in previous studies of SOLEIL^14^. *ω*_*s*_/2*π* is the synchrotron frequency (not to be confused with the storage ring revolution time), and *τ*_*s*_ is the synchrotron damping time. *c* is the speed of light in vacuum and *η* measures the dependence of the round-trip time with the electron energy. *η*_*N*_*ξ* is a gaussian white noise term, with 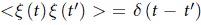. Parameter values are given in the Methods section.

Typical numerical results are presented in Fig. 5. When the electron bunch charge exceeds a threshold, finger-like structures are spontaneously formed in the electrons phase space (Fig. 5a). Furthermore, the whole electron bunch distribution experiences a global rotation near the so-called synchrotron frequency (4.64 kHz here), and evolves in a bursting and irregular way.

The longitudinal electron bunch shape (Fig. 5b) is deduced from the vertical projection of the phase space distribution (Fig. 5a). Then the CSR THz field at the electron bunch location (Fig. 5c) is deduced from the electron bunch shape (Fig. 5b). As can be seen in Fig. 5c, only the fast variations lead to a significant coherent terahertz field. This natural “AC-coupling” is an advantage for the observation as it removes the global (slow) electron bunch shape, and let pass only the important information. Because the electron bunch distribution rotates counter-clockwise in phase-space (see Fig. 5a and supplementary movie), the microstructures drift along the longitudinal position toward the head of the electron bunch (Fig. 5c). The drift of the structures (in Figs. 4 and 5d) can thus be interpreted as a consequence of the formation of “fingers” in the lower part of phase space (Fig. 5a). Main features of the theoretical predictions are found in relatively satisfying agreement with the new experimental findings.

In conclusion, the present time-stretch strategy allows one to perform a “time-lapse observation” of microscopic structures that appear within charged relavististic objects. The advantages over classic single-shot electro-optic sampling strategies are a simultaneous improvement on both the aquisition rate and the sensitivity. Such quantitative studies open-up to a new level of understanding of electron beam dynamics, and severe tests of theoretical models. We believe that this strategy may be a key contribution in situations where high acquisition rate measurements are needed. Straightforward applications concern the investigation of the THz pulses emitted by other storage rings, and by terahertz free-electron lasers. The technique can also be transfered to high-repetition rate linear accelerators, provided an electro-optic sampling system can be used^18^. Perspectives in ultrafast spectroscopy are also envisaged, as the instantaneous THz spectrum can be straightforwardly deduced from the electric field shape (inset of Fig. 3). In addition to the THz domain, the present time-stretch strategy also opens new possibilities at short wavelengths. Important perspectives to be explored concern the monitoring of optical pulses from high-repetition rate EUV and X-ray Free-Electron Lasers, for instance by associating the time-stretch strategy to transient reflectivity setups^30^,^31^.

## Methods

### Ultrafast recording setup

The detailed setup is presented in Fig. 6. It exclusively uses off-the shelf components, and is composed of three parts:

- A classical system for generation of chirped laser pulses, using a femtosecond laser and a dispersive fiber.
- A classical electo-optic modulation system, based on the Pockels effect in a GaP crystal.
- A specially designed balanced time-stretch device. This setup disperses the optical pulses up to the nanosecond range. Thus we can achieve simultaneously a high repetition rate, and at the same time a high sensitivity thanks to the possibility of the balanced detection. This is the key point of the setup.

### Production of the stretched laser pulses

We use a femtosecond Ytterbium-doped fiber laser (Orange) from MENLO GmbH. The emitted pulses have a spectral bandwidth of 50 nm, and the total output average power is 40 mW. The repetition rate is chosen at 88 MHz, which corresponds to 1/4 *th* of the RF frequency of Synchrotron SOLEIL and 104 times the electron revolution frequency. It is synchronized on the RF clock of the storage ring using a RRE-Synchro system from MENLO GmbH.

The pulses are chirped by a polarization-maintaining fiber (PM 980), which length determines the temporal window of the acquisition (typically few tens of picoseconds). The length *L*_*1*_ which is used in the calculation of the stretch factor *M* = 1 + *L*_2_/*L*_1_ is the sum of two components: the actual length of the fiber used after the laser (10 m here), and a small contribution due to pulse dispersion inside the laser (estimated to 0.7 m of propagation in a PM980 fiber). Thus we took *L*_1_ _=_ 10.7 m, leading to *M* ≈ 190.

A polarizer is placed at the output in order to remove possible spurious components with the wrong polarization.

### Electro-optic modulation device

**Figure 2:**
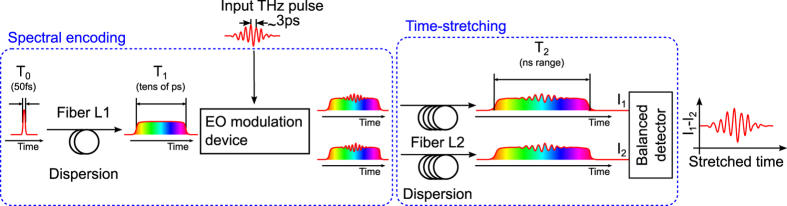
Principle of the photonic time-stretch device realized for slowing down the information, while keeping a high sensitivity. The THz pulse under investigation is first encoded into a chirped laser pulse, using an electro-optic crystal (“EO modulation device”). This device (see Fig. 6 for detils) provides two complementary outputs. Then the optical information of each output is simply stretched from picoseconds to nanoseconds by propagation in a long fiber (2 km). Balanced detection is performed between the two stretched laser pulses, thus providing a very high sensitivity for the device by removing the “DC” background. Details of the optical system are presented in the Methods section.

**Figure 6:**
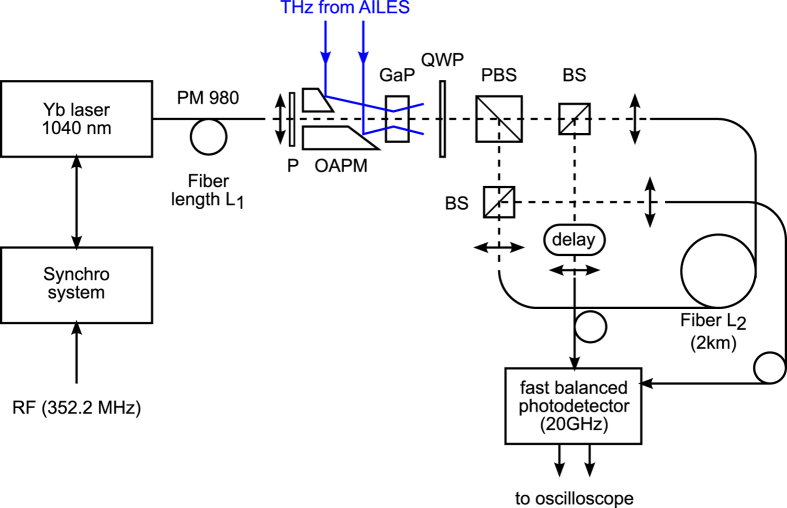
Experimental setup for recording the THz pulses at 88 MHz acquisition rate. P: polarizer, OAPM: off-axis (gold-coated) parabolic mirror. GaP: Gallium Phosphide crystal. QWP: achromatic quarter-wave plate. PBS: polarizing beam-splitter. BS: beam-splitter with low polarization-dependent losses. The 2 km fiber is an HI1060 from Corning. All fiber collimating and focusing lenses have 11 mm focal length. (Delay) is a delay line, allowing to adjust the relative delay between the two balanced photodetector inputs.

This part corresponds to the *EO modulation device* in Fig. 2 and from (P) to (PBS) in Fig. 6. The THz radiation available at the focusing point of the beamline is first collimated using an off-axis parabolic mirror (not shown in Fig. 6), with 101.6 mm focal length. It is then focused in the GaP crystal using an off-axis gold-coated parabolic mirror (OAPM in Fig. 6) with 50.8 mm focal length, and a 3 mm hole. The laser and the THz radiation interact in a [110]-cut GaP crystal with 5 mm length (10 × 10 × 5 mm^3^). The [-110] axis is parallel to the polarizations of the laser and the THz beam. An achromatic quarter-wave plate (QWP) is inserted after the GaP crystal. Its optical axis oriented at *π*/4 with respect to the laser polarization. Finally, a polarizing cube beam-splitter (PBS) provides the two outputs of the EO modulation device. At the outputs of the PBS, the pulses contain an intensity modulation which is a “replica” of the THz pulses, and the two outputs are modulated in opposite phase.

### Time-stretching of the two optical pulses, using a single fiber

Instead of using physically different fibers for the final dispersion (Fig. 2), we use a variant that is much more robust from the experimental point of view. As can be seen in Fig. 6, the two output pulses of the polarizing cube beam-splitter are sent in the same fiber, in opposite directions. Finally, two beam-splitters (BS) extract the two pulses, which are sent to a fast balanced photodetector. This technique (which reminds the idea of the Sagnac loop) permits to obtain the same path on the two laser pulses even when the fiber optical length fluctuates.

The *L*_2_ fiber is an HI1060 from Corning with 2 km length (and an overall attenuation of the order of 3 dB). and the beam-splitters are chosen to have low polarization-dependent losses (Newport 05BC17MB.2). This choice for *L*_2_ leads to stretched pulses of ≈4.5 ns, and 1.35 mW peak power is typically dectected in each balanced photodetector channel input.

### Recording electronics

The detection and subtraction between the two stretched signals is performed using a DSC-R412 InGaAs amplified balanced photodetector (photoreceiver) from Discovery Semiconductors, with 20 GHz bandwidth and 2800 V/W gain (specified at 1500 nm). The two differential outputs of the detector are sent on a Lecroy LabMaster 10i oscilloscope with 36 GHz bandwidth, 80 GS/s sample rate on each channel, and a memory of 256 Mega samples.

### Data processing

In absence of THz signal, the recorded balanced signal presents a non-zero shape which corresponds to imperfections of the setup, in particular small polarization dependent losses (that depend on wavelength). Since this “background” signal is deterministic (i.e., is the same for each laser pulse), it is easly removed from the signal, by a simple subtraction.

### Transport of the terahertz beam

We operated the time-stretch setup at the A branch of the AILES beamline, just before the interferometer (see Ref. 32 for the beamlline detail). The focusing point was imaged onto the GaP crystal, using a telescope composed of a 101.6 mm focal length off-axis parabolic mirror (not shown in Fig. 6) and a 50.8 mm off-axis parabolic mirror (OAPM in Fig. 6).

### Perfomances of the setup

The special setup presented in Fig. 6 provides higher acquisition rates (in terms of number of pulses per second) than classical spectral encoding methods. The reason is that oscilloscopes can nowadays reach much higher data acquisition rates (80 Giga samples/s here) than the cameras which are necessary for spectral encoding. Here, the acquisition rate capability is limited by the repetititon rate of the laser, namely 88 MHz.

At the same time, the setup allows us to perform acquisition with higher sensitivity than the traditional single-shot electro-optic sampling, because, of the possibilty to achieve balanced detection at the analog level. The sensitivity is here mainly limited by the noise of the amplified balanced detector. The RMS noise on the finally recorded signal can be easily measured, and this gives a measure of the system sensitivity. The RMS noise (over the 20 GHz bandwidth of the phototetector) corresponds to a birefringence-induced phase shift in the GaP of 3.2 × 10^−3^ Radian. Assuming that the relevant electro-optic coefficient of GaP is *r*_41_ = 0.97 pm/V, and neglecting the THz frequency dependence of *r*_41_, the sensitivity is estimated to be 37 V/cm (inside the crystal) near the laser pulse peak.

The SOLEIL storage ring was operated in single bunch, normal alpha mode, with natural bunch length *σ*_*z*_ = 4.59 mm, relative energy spread *σ*_*δ*_ = 1.017 × 10^−3^, and a momentum compaction factor *α* = 4.38 × 10^−4^. The ring was operated at a current of 15 mA, which is above the microbunching instability threshold (≈10 mA). Other parameters are described in Ref. 14. The model parameters *η* and *η*_*N*_ are defined by 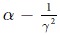 and 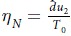, with *T*_0_ the storage ring revolution time.

## Additional Information

**How to cite this article**: Roussel, E. *et al*. Observing microscopic structures of a relativistic object using a time-stretch strategy. *Sci. Rep.* **5**, 10330; doi: 10.1038/srep10330 (2015).

## Supplementary Material

Supplementary Video

## Figures and Tables

**Figure 1:**
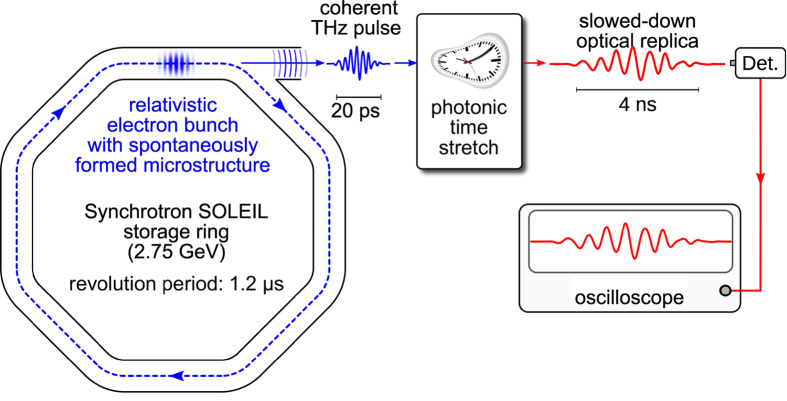
General principle of the experiment. A relativistic electron bunch circulating in the SOLEIL storage ring (2.75 GeV) presents microstructures, that evolve in a complex way. The coherent THz radiation emitted at a bending magnet carries the information on the microstructure shape, but is too fast (at picosecond scale) to be recorded by traditional means. The present strategy consists in “slowing-down” the information in order to obtain an optical replica at the nanosecond scale, so that a conventional oscilloscope can be used for the recording. The soft clock has been adapted from https://openclipart.org/detail/16605/clock-sportstudio-design-by-fzap (public domain CC0 1.0 Universal).

**Figure 3:**
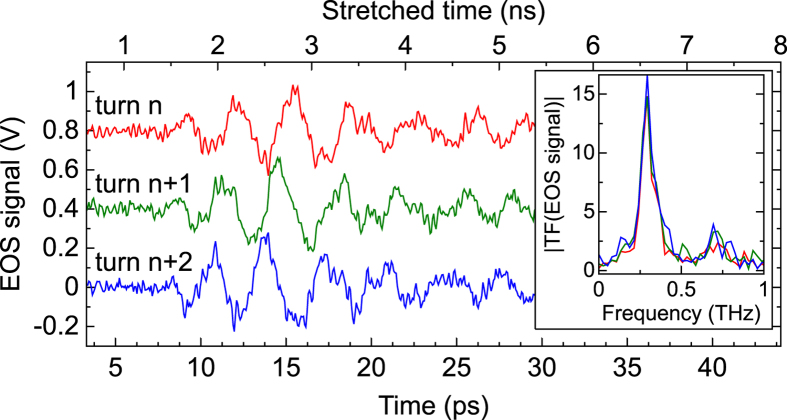
Typical single-shot recordings of coherent THz pulses, which carry the information on the electron microstructure. The lower scale corresponds to the real time of the phenomenon. The upper scale corresponds to the time at the oscilloscope input (i.e., after the photonic time stretch by a factor 190 ). The time between pulses is of the order of 1 *μ*s, but the system is actually recording one signal every 12 ns. The effective A/D conversion speed is 15 Tera samples/s. Inset: Power Fourier spectra of the three pulses.

**Figure 4:**
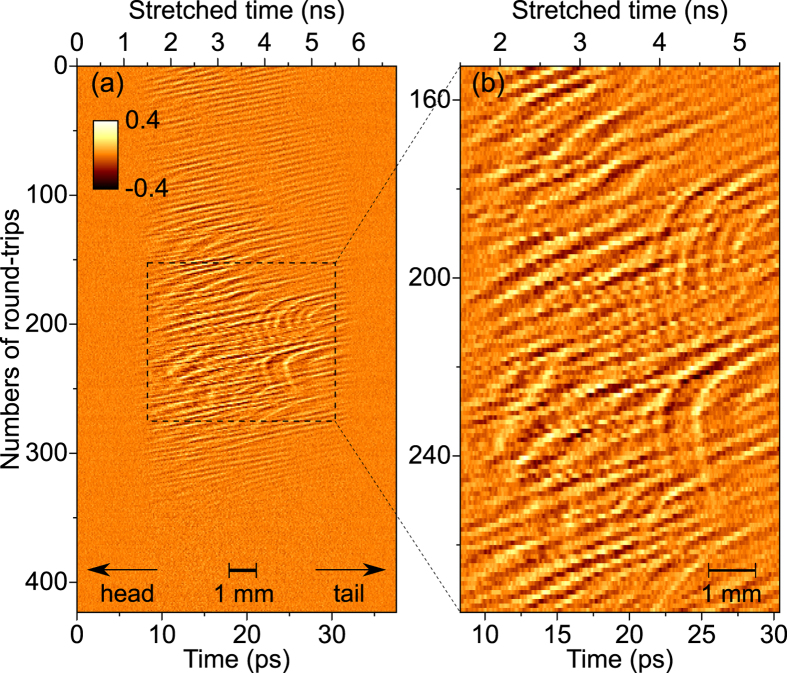
Recorded CSR pulses (i.e., containing the information on the electron bunch longitudinal modulation) versus the number of round-trips in the storage ring. (**b**) is a zoom on the rectangle part in (**a**).

**Figure 5:**
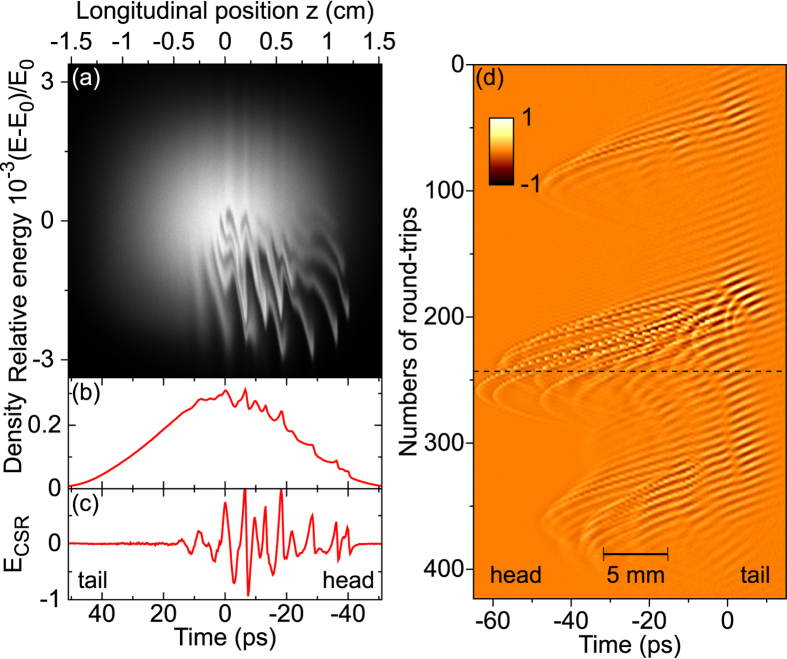
Numerical simulation of the relativistic electron bunch dynamics. (**a**) Distribution of the electrons in position-energy phase space (1.1 × 10^8^ particles are taken into account in the simulation). The pattern globally rotates at a slow frequency near 4.64 kHz, and evolves in a complex way (see supplementary movie). (**b**) Electron bunch profile (i.e., the projection of (**a**) onto the *z* axis), and (**c**) CSR field. (**d**) evolution of the CSR pulse shapes versus number of round-trips. The dashed line corresponds to the (**c**) pulse.
